# Low-Cost 3D Printer Drawn Optical Microfibers for Smartphone Colorimetric Detection

**DOI:** 10.3390/bios12020054

**Published:** 2022-01-19

**Authors:** Md Arafat Hossain, Protik Chandra Biswas, Saptami Rani, Shinthia Binte Eskender, Md Foyez-ul Islam, Arbil Chakma, John Canning

**Affiliations:** 1 Department of Electrical and Electronic Engineering, Khulna University of Engineering & Technology, Khulna 9203, Bangladesh; protik@eee.kuet.ac.bd (P.C.B.); rani2003752@stud.kuet.ac.bd (S.R.); tanveerfoyez@gmail.com (M.F.-I.); arbilchakma13@gmail.com (A.C.); 2 Department of Materials Science and Engineering, Khulna University of Engineering & Technology, Khulna 9203, Bangladesh; sinthiaoyshi@gmail.com; 3 School of Chemistry, University of Sydney, Sydney, NSW 2006, Australia; 4 Laseire Consulting Pty Ltd., Sydney, NSW 2006, Australia

**Keywords:** 3D printing, polymer optical fiber, thermal extrusion, polyethylene terephthalate glycol, fused deposition modeling, smartphone sensor, lab-in-a-phone, extra virgin olive oil

## Abstract

A fused deposition modeling (FDM) 3D printer extruder was utilized as a micro-furnace draw tower for the direct fabrication of low-cost optical fibers. An air-clad multimode microfiber was drawn from optically transparent polyethylene terephthalate glycol (PETG) filament. A custom-made spooling collection allows for an automatic variation of fiber diameter between *ϕ* ∼ 72 to 397 μm by tuning the drawing speed. Microstructure imaging as well as the 3D beam profiling of the transmitted beam in the orthogonal axes was used to show good quality, functioning microfiber fabrication with uniform diameter and identical beam profiles for orthogonal axes. The drawn microfiber was used to demonstrate budget smartphone colorimetric-based absorption measurement to detect the degree of adulteration of olive oils with soybean oil.

## 1. Introduction

Three-dimensional (3D) printing is a disruptive additive manufacturing technology that offers cost-effectiveness and inherent design flexibility. Excitingly, it is beginning to influence optical fiber fabrication, novel waveguides and functional materials [1,2,3,4,5], potentially offering consumer access to what are traditionally expensive technologies [6]. Photonics applications are particularly interesting given the extraordinary efforts over the last few decades to advance fibers customized for new diagnostic and Internet of Things (IoT) applications, varying not only in composition but in structure as well. As the processes involved with optical fiber fabrication more broadly are better understood [7], it is clear that 3D printing of optical fiber preforms will disrupt their traditional counterparts involving stack-and-draw capillary [8,9] and chemical vapor deposition [10], for example. 

Among the available 3D printing methods, fused deposition modeling (FDM) technology is widely accessible, fast and low-cost writing [11]. FDM shows more promise for producing commercial grade machinery parts with high density and less bubbles, whereby polymer filament material is extruded through a hot nozzle and deposited line-by-line and layer-by-layer to produce a 3D scaffold. Initially, 3D-printing in photonics began with the fabrication of polymer optics using FDM printers [12,13], because of the low extrusion temperature of desktop printing, whilst most recent works demonstrated the fabrication of silica fiber [14]. The process offers great flexibility for structural fiber fabrication although it still relies on the traditional drawing process to fabricate the final fiber from the preform [12,13,14,15]. This drawing process is expensive, especially for application-specific optical fibers where volume demand is not there to bring costs down. For this reason, the wider deployment of traditionally produced optical fiber in the IoT beyond communications has been slow.

An important realization has been that the drawing process is fundamentally not linked to size—that is provided the thermal control, both of heating and cooling, in the process is high, the extrusion chamber for produced and drawing optical fiber is not size dependent. For example, currently, 3 m tall polymer fiber draw towers can in principle be replaced with micro-heaters and pulley systems to produce high quality optical fiber. In particular, the temperature distribution and control of even a low budget desktop FDM printer extruder is so good that it can act as a high-quality micro draw tower for producing optical fiber. We have previously characterized with high precision the thermal profile of the extruder head using a silica optical fiber Bragg gratings (FBGs) temperature sensor [16]. Based on this work, polymer air-clad fibers as thin as 30 μm was drawn. This has also opened the possibility of drawing fiber with cores from a specially designed extruder head [17]. Later, inspired by this work, other colleagues demonstrated chalcogenide glass filaments of 1.75 mm diameter, fabricated from a preform of the same material which is then used to draw glassy fiber using the direct extrusion on a FDM printer [18]. The layer-by-layer deposition using FDM printer has been used to additively fabricate optics such as air-clad fiber optic faceplates [19], lenses [20,21], gratings [22], cuvettes [23] and many more. The 3D-printed element has been widely accepted and utilized in many sensory applications. For example, 3D-printed polymer packages have been embedded to protect long period fiber Bragg gratings (LPG) stress and temperature sensors [24,25,26,27] and tapered optical fiber for humidity and gas sensing [28]. Helical distributed feedback FBG and rocking filters have also been written in a fibers fabricated from a 3D printed glass preform [29].

Despite tremendous progress in the last decade, 3D-printed optics are found to have limited performance in the visible spectrum with many applications focused in the terahertz region [3,20,30,31]. Due to the high attenuation of polymers optics, low printing resolution, and noticeable surface roughness between successive layers, the 3D-printed object requires additional post-treatment of materials such as thermal annealing [32] and acetone vapor smoothing before use within an application [33,34]. These methods often change the optical properties and geometry of the final object making preemptive high accuracy design challenging. The physical properties of the fiber also depend on the printing process and orientation of the layers and need to avoid delamination [35]. High quality optics with low attenuation and highly flexible and durable materials with low-cost fabrication techniques are significantly important to fill this gap. The utilization of the printer’s thermal extrusion for direct drawing of highly durable and flexible polyethylene terephthalate glycol (PETG) materials through the hot end at a suitable temperature can produce high quality optical fibers. The inclusion of automatic control allows considerable flexibility and tuneablity in precision design.

One area that can benefit tremendously from 3D-printed low-cost customized elements is various field-portable analytics or diagnostics, including wearables and biosensor-edge devices in the IoT, in which customized, flexible, user friendly operation with enhanced detection capabilities, high throughputs and improve signal-to-noise ratio are required [36,37,38]. An area of recent interest is the integration with smartphone technologies to enhance their sensing capabilities [39,40]. Currently, 3D-printed enclosures are used in most of the smartphone-based biomedical instruments to keep the hardware components in their fixed position and block stray light illumination [41,42,43]. Specially designed 3D printed optics such as lenses, micro-prism arrays, diffusers and beam splitters have been used in many applications [44,45]. The use of optical fibers can enhance light collection and increase flexibility in various endoscopic applications as well as reach samples that are normally difficult to reach—a degree of remote interrogation is possible [46,47]. In this work, we demonstrate the direct drawing of air-clad optical microfibers from an extruder head and use these in a smartphone optical fiber sensing application. Electronic control of the 3D-printed fiber drawing allows precise tuning of the diameter of the drawn fibers. The fiber samples have been characterized for microstructure measurements and axial deviations during propagation of light. The drawn microfiber is well suited to chemical sensing and collecting light in endoscopic-style applications. As a proof of concept, optical fiber-based smartphone colorimetric sensing of olive oils is demonstrated. Using disposable sensing components that take detection into the field greatly expands current gold-standard colorimetric diagnostics for detecting various species within a range of biomedical, agricultural, and environmental samples.

## 2. Microfiber Drawing System

**Figure 1:**
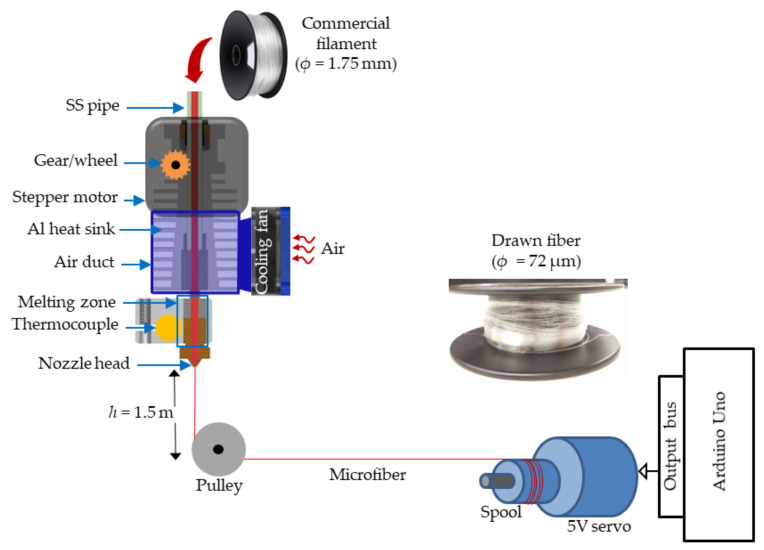
Schematic showing the microfiber drawing process using thermal extrusion from an FDM 3D printer nozzle with an automated fiber spinning collection system.

The 3D printer used in this fiber drawing process is an FDM Zortrax Inventure (Zortrax S.A, Olsztyn, Poland) working with “layer plastic deposition (LPD) plus” technology. This model was chosen because of its precise temperature profile and high temperature (*T*_max_ = 380 °C) extrusion capacity that covers a wide range of printable materials, from simple polylactic acid (PLA) and acrylonitrile butadiene styrene (ABS) to different thermoplastic polyurethane filaments (TPU) such as polyethylene terephthalate (PET), polyethylene terephthalate glycol (PETG), with good optical properties. It is equipped with two extruders (Extruder 1 and 2) dedicated for printing the model and of support material although the single extruder structure without any modification in its temperature profile (shown schematically in Figure 1) has been used for the fiber-drawing process. For most of the 3D printers designed for flexible polymetric filaments, the filament feeding process is performed with a gear wheel shafted with a 24 volts stepper motor. The filament is then passed through an aluminum heat sink channel to the heating zone where it is melt down by resistive heating and dynamically pushed through a circular nozzle head of diameter *ϕ* = 0.4 mm. A cooling fan fitted with an aluminum heat sink increases thermal isolation between the hot zone and plastic feeding element. The melted polymer is pulled down by a pulley system located underneath the nozzle head at *h* = 1.5 m and collected to a metal gear shafted spool (*ϕ* = 20 cm) with a servo motor (N20 DC 6 V 200 rpm) with continuous rotation. The speed of the pulling system and speed of the feeding material determine the fiber diameter. To automate control of the fiber diameter, a microcontroller (Arduino Uno)-based voltage control is used to control the speed of the pulling system which offers low-cost and easy diameter control. PETG microfibers with different diameters were drawn by varying the speed of the motor spool. To achieve the appropriate polymer viscous flow for PETG, the extrusion temperature was set at *T*_ex_ = 240 °C. A nozzle diameter of *ϕ* = 0.4 mm and 100% flow rate of filament was used during printing. Consistent diameter control was achieved by keeping the nozzle head fixed in the XY-plane during drawing. The printing chamber and feed material chamber are sealed off with a small opening fiber to exit, protecting the process from the environment and stabilizing 3D printing conditions. Additionally, a high efficiency particulate air filter was in-built with the printer prevents smoke and soot from interfering with the fiber output as well as to ensure safe conditions.

## 3. Materials and Characterization

The microfiber was drawn from Z-GLASS translucent filament (*ϕ*_F_ ∼ 1.75 mm, price ∼ 49.00 USD/800 gm) purchased from Zortrax. Its main constituents are PETG ∼ 80%, fiberglass filings = 8∼12%, additives and colorants = 0∼8%. PETG is a good choice for polymer optical fiber which adds glycol with standard PET material to increase the tensile strength and eliminate brittleness over bending in various flexible applications. High durability and low shrinkage after printing of the PETG material make them suitable for applications in extreme environments and resistant to oils and greases. PETG itself has good optical transparency, although it can vary depending on the constituents.

**Figure 2:**
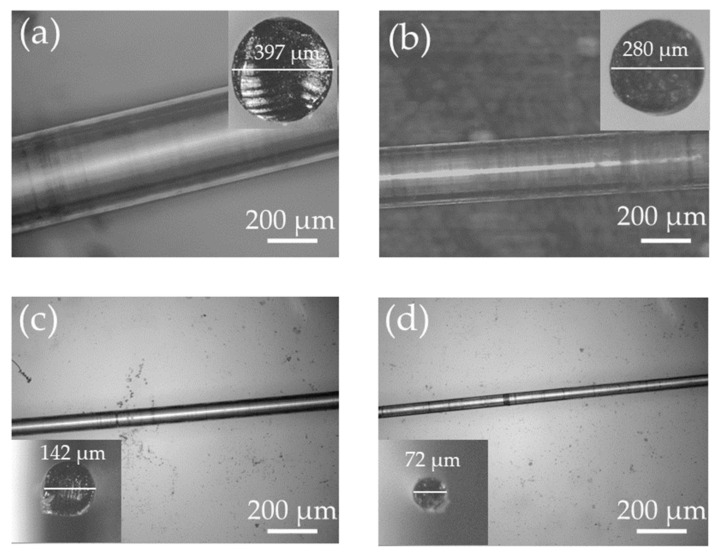
Microstructure images of the 3D-printed fibers drawn at different drawing speeds. (**a**) Without external weight (gravitational pulling); (**b**) 50 rpm; (**c**) 150 rpm and (**d**) 180 rpm of the motor speed. 100% flow rate of filament is used in all cases.

The PETG microfiber samples drawn by different spooling speeds were first washed in isopropanol and dried in air for *t* = 24 h. A hot knife edge was used to cleave the microfiber. Fiber samples with different diameters were imaged under an optical microscope, shown in Figure 2. The microfiber with the largest diameter, *ϕ*_max_ ∼ 397 μm, was achieved at a 100% flow-rate of filament and only by gravitational pulling, i.e., without applying any applied external weight (Figure 2a). The fiber with the smallest diameter, *ϕ*_min_ ∼ 72 μm, was drawn at 180 rpm of the motor speed in addition to the gravitational pulling and with the same flow rate of the filament (Figure 2d). The maximum *ϕ* of the fiber is determined by the size of the nozzle diameter (in this case *ϕ* = 0.4 mm) while minimum *ϕ* is determined by the composition of the material and viscosity of the polymer.

**Figure 3:**
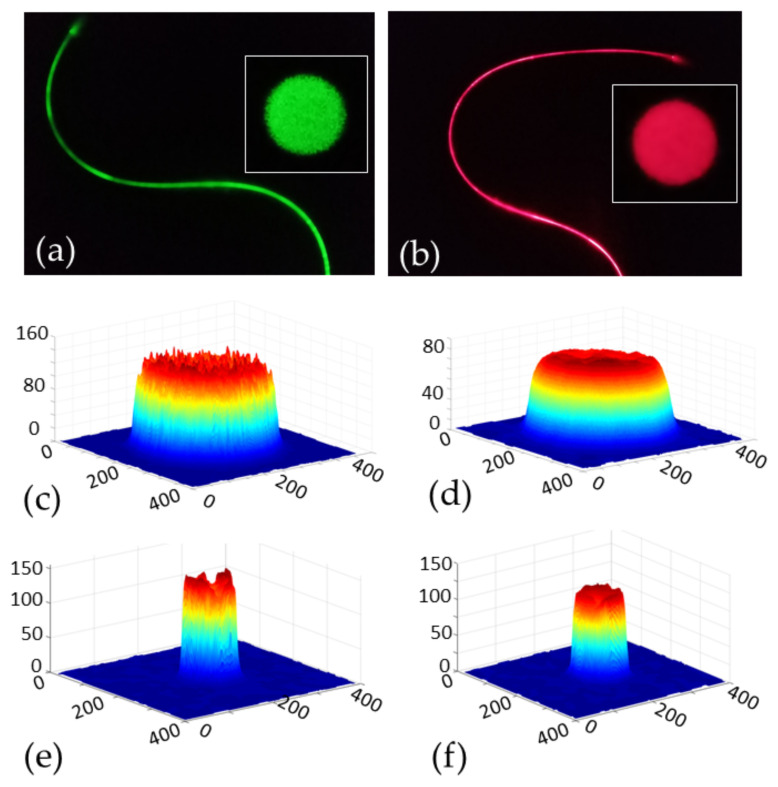
Beam profile characteristics to analyze the axial deformation of the drawn microfiber of different diameters: (**a**,**b**) photographs of green and red laser light guided through the microfiber; (**c**–**f**) corresponding 3D beam profile over the *x–y* plane measured in terms of pixel numbers of the smartphone CMOS camera system/detector. (**c**,**d**) beam profile for *ϕ* ∼ 397 μm and (**e**,**f**) beam profile for *ϕ* ∼ 142 μm.

To study the effect of thermal extrusion on the structural deformation of the fiber cross-section as well as the guiding modes of light wave, beam profiles of the multimode fibers were taken. They were analyzed using a novel smartphone based 3D beam profiler [48]. Monochromatic emissions of two laser pointers *λ*_green_ ∼ 532 nm (green) and *λ*_red_ ∼ 650 nm (red) were passed through a section of the microfiber (*ϕ* ∼ 397 μm, *L* = 50 cm). The output beam at the end of the fiber was projected on the surface of a plane white paper placed orthogonally to the propagation direction. The projected beam was imaged on a smartphone camera and processed for 3D spatial profiling on a smart application (app) software. The app plotted the spatial dimension in *x* and *y* axes, whereas the intensity was plotted on the z-axis. The beam diameter at different axis was calculated to determine the variation between different axes on the *xy*-plane. For the fiber of largest diameter, the beam width ratio was found to be *ϕ*_x_/*ϕ*_y_ ∼ 0.974 ± 0.05 and 1.003 ± 0.02, respectively, for the green and red light coupled into the fiber Figure 3c,d. This indicates good circular concentricity of the fiber cross-section. Relatively larger levels of deformation were observed in the smaller fiber as also noticed in the microscope images in Figure 2. This deformation may have been introduced from the asymmetrical tension on the fiber radial axes because of the vertical inclination (<5 degree) of the 3D-printer extruder with respect to the underneath spool collection. For the fiber *ϕ* ∼ 142 μm, the ratio was found as *ϕ*_x_/*ϕ*_y_ ∼ 0.982 ± 0.10 and 1.018 ± 0.07, respectively, for green and red light as shown in Figure 3e,f. Nevertheless, the profiles show highly multimodal propagation of light through the microfibers due to large and uniform scattering centres throughout the fiber geometry. Relatively larger scattering was noticed at lower wavelength in both cases as shown in Figure 3.

## 4. Absorption Based Sensing of Olive Oil Using 3D-Printed Fiber

We previously reported on the visible transmission spectrum of 3D-printed PETG materials, which was consistent with other reports [12,13]. Strong attenuation was observed in the near-UV and blue region below *λ* ∼ 400 nm. Above this wavelength, the transmission spectrum was found to be nearly flat. The measured attenuation (α = 0.26 dB/cm) using a green laser at *λ* = 543 nm was comparable with commercial grade polymer optical fiber produced by other means [16]. The optical air-clad fibers we produced here are cheap and suited for low-cost sensing in the field, and potentially disposable. Optical fiber-based sensing can benefit agricultural sensing, particularly in assessing food quality [49]. Examples in which fibers have been used include fiber-coupled external sensors taking measurements based on optical methods such as absorption, fluorescence, Raman scattering, evanescent wave, and surface plasmon based techniques. Here, visible absorption measurements of vegetable oils using the produced fiber were demonstrated. Extra virgin olive oil is distinguished from soybean oil.

**Figure 4:**
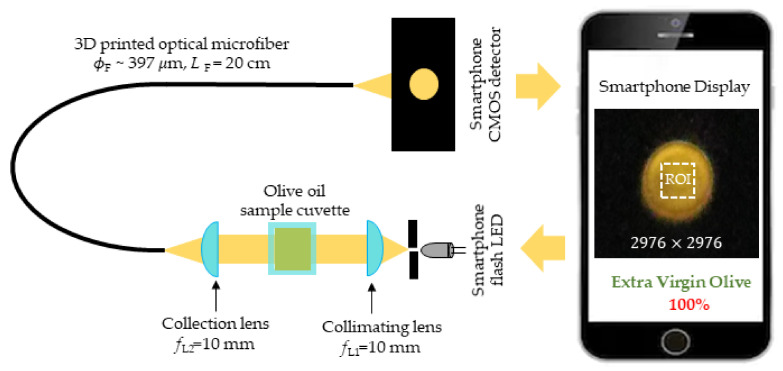
3D-printed air-clad optical microfiber based visible light absorbance measurement of olive oil. Smartphone camera detection demonstrates field-portable measurement and reporting.

A smartphone in-built flash LED was used as a broadband optical source over the visible spectrum (*λ* = 400 ∼ 700 nm). The source output was collimated using a pinhole (*ϕ* = 2 mm) and collimating lens (*ϕ* = 25 mm, *f*_L1_ = 10 mm) and transmitted through the oil samples contained in quartz cuvettes (1 cm × 1 cm × 5 cm). The transmitted light was collected using a second collimating lens (*ϕ* = 25 mm, *f*_L2_ = 10 mm) into the input end of the microfiber (*ϕ* ∼ 397 μm, *L* = 20 cm) through which the light propagates and finally reached the smartphone camera detector. The camera produced an image of the output end of the microfiber containing the color information of the sample at a distance *l* = 5 cm front to the rear facing camera unit of the smartphone. Figure 4 summarizes the setup. The smartphone camera has been used widely for the colorimetric variation of optical signal at its red, green, and blue (RGB) color channels. The CMOS camera of a smartphone uses a Bayer filter in a periodic pattern of RGB color filters, which separate the color information spectrally at three different spectral bands for red, green, and blue channels [50]. The spectral responses are camera dependent and significantly vary from model to model but a device specific calibration can be applied to use it directly for colorimetric sensing [44].

The vegetable oil samples were collected from a local supermarket. They include extra virgin olive oil (EVO—imported from Borges, Spain) and soybean oil (produced locally in Bangladesh). Soybean oil is a widely consumed, inexpensive vegetable oil available locally, whereas olive oils are considered as a healthy item due to the presence of essential monounsaturated fatty acids, vitamins (A and E) and carotenes and usually imported from overseas suppliers. Reasonably, the latter is considered as a premium item with a high price. The quality of EVO can often degrade during manufacturing, packaging, transportations, and storage. It is also highly likely that the EVO items are mixed with ordinary vegetable oils such as soybean oil. To detect the presence of soybean oil in EVO, a total of five test samples—100%, 75%, 50% and 25% EVO oil and 100% soybean oil were prepared by varying their relative volume within the fixed sample volume *V* = 5 mL. To avoid any possible photo–and thermal degradation, the oil samples were stored in a dark bottle at room, with temperature, *T* ∽ 22 °C.

**Figure 5:**
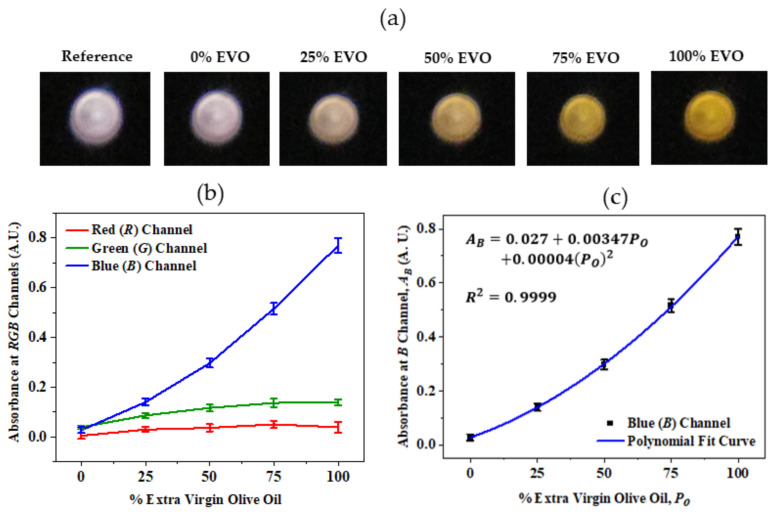
The results of absorbance measurement vegetable oil samples at the RGB color channels of the smartphone camera: (**a**) photographs of the microfiber output end for different samples; (**b**) absorbance at RGB color channels vs. percentage of extra virgin olive oils in sample and (**c**) polynomial fit of response measured at the blue channel. There is a slight decrease in red absorption in part due to some red fluorescence from absorbed blue [47].

To measure the absorbance of oil samples at the RGB color channels, the light signal collected using the microfiber from oil samples were imaged onto the smartphone camera as shown in Figure 5a. The measurement was performed in a dark room to ensure no ambient illumination reached the detector. A 3D-printed enclosure can be used as in other smartphone studies, cuts ambient light and is also suitable for portable field operations. To eliminate the absorption due to PETG microfiber as well as other elements in the system, a reference measurement was performed for a cuvette with no sample (Figure 5a). The absorbance of a sample at the red channel (*A_R_*) was obtained by measuring the average red pixel intensity (*R*) within a fixed region of interest (ROI) of 100 × 100 pixels of sample (*R_s_*) and reference (*R_r_*) solutions and finally applying them in the Beer-Lambert equation [*A_R_* = log(*R_s_/R_r_*)]. The absorbance at other color channels (*A*_G_ and *A*_B_) were also determined in a similar way and plotted in Figure 5b. The results show the strongest absorption at the blue channel and weakest absorption at red channel in EVO oils compared to other channels that satisfy the absorption spectrum measured using standard benchtop instruments in our previous work [39]. This assumes that the amount of red fluorescence that may occur with blue absorption is comparatively small. These absorption increases with the increase in the percentage content of olive oil (*P*_o_) in the samples. This *A*_B_ is primarily attributed as the absorbance due to *β*-carotene which is a source of pro-vitamin A and acts as an antioxidant in olive oil. The weak absorption in the red channels can be attributed to absorption due to chlorophyll. However, the measurement of EVO from *A*_R_ is highly affected by the red fluorescence generated within the sample due to blue excitation. A polynomial fit relating *A*_B_ with *P*_o_, as shown in Figure 5c, can be used directly to determine the percentage content of EVO in an unknown sample.

## 5. Conclusions

In summary, we demonstrated the potential of a low-cost desktop printer to have excellent micro-furnace extrusion heads suitable for drawing optical fiber directly. This technology can be a low-cost alternative for the direct fabrication of optical elements that are useful for various sensing applications with an improvement in detection capacity, design flexibility and user accessibility. The microstructure investigation shows good quality polymer fiber optical fibers from highly durable PETG filament with uniform diameter and highly multimode propagation of visible wavelengths. The drawn microfibers were utilized for endoscopic applications in smartphone colorimetric sensing. The use of 3D-printed optical elements can avoid the need for high end commercial grade optics and packaging components in different instruments available for biosensing. The direct drawing of a microfiber using the thermal extrusion technology creates significant room to scale up, for example, by fabricating single and multi-core fibers from structured filaments or nozzle heads. Automatic control of fiber diameter can be useful for fabrication of tappers with desired size.

As an application of the drawn microfiber, a smartphone optical fiber colorimeter has been investigated for the detection of extra virgin olive oils adulterated by soybean oil. The use of optical fiber increases flexibility as well as signal-to-noise ratio suitable for applying the technique for endoscopic detection and chemical analysis in fields. The instrument can be used to determine arbitrary compositions within a bottle or container and can ensure quality assurance is being met in the field. An artificially processed or adulterated produce is therefore straight forward to identify and report immediately. Ideally, 3D printing technology can be used at any place for fabricating all parts of the measurement system, including a customized collection lens, slit, diffuser, cuvette, and the entire package holding all optical components. This is ideal for democratizing technology via global accessibility and large-scale deployment.
